# Safety and efficacy of a temperature-controlled ablation system for ventricular tachycardia: Results from the TRAC-VT study

**DOI:** 10.1007/s10840-025-01995-z

**Published:** 2025-02-01

**Authors:** Josef Kautzner, Javier Moreno, Claudio Tondo, Frédéric Anselme, James Burrell, Daniel Becker, Petr Peichl, Ian Patchett, Tarvinder Dhanjal

**Affiliations:** 1 Institute for Clinical and Experimental Medicine, Prague, Czechia; 2 Department of Cardiology, Hospital Universitario Ramón y Cajal, Madrid, Spain; 3 Dept of Clinic. Electrophysiology & Cardiac Pacing, Heart Rhythm Center, Centro Cardiologico Monzino, Milan, Italy; 4 Cardiologie, CHU de Rouen, Rouen, France; 5 Medtronic, Minneapolis USA; 6 Department of Cardiology, University Hospitals Coventry & Warwickshire NHS Trust, Coventry, UK; 7 Heart Rhythm Research Group, Division of Biomedical Sciences, Warwick Medical School, Clinical Sciences Research Laboratory, Coventry, UK; 8 Department of Biomedical, Surgical and Dental Sciences, University of Milan, Milan, Italy

**Keywords:** DiamondTemp, Cardiac arrhythmia, Radiofrequency catheter ablation, Temperature controlled, Ventricular tachycardia

## Abstract

**Background:**

Catheter ablation using radiofrequency (RF) energy is an established treatment for ventricular tachycardia (VT). Tissue temperature is a key determinant of successful lesion creation, and yet, it is difficult to measure during conventional RF ablation because of the cooling effect of high-flow rate saline irrigation. The TRAC-VT study evaluated the safety and efficacy of a novel irrigated RF ablation system modulating power based on real-time tissue temperature.

**Methods:**

Patients with sustained monomorphic VT and structural heart disease (SHD) were enrolled. Catheter ablation was performed in temperature-control mode (irrigation 8 ml/min, temperature set-points 55 or 60 °C, and power output ≤ 50 W), with RF applications for ≤ 45 s. The primary safety endpoint was a composite of cardiovascular-specific serious procedure-related adverse events within 30 days post-ablation. The primary effectiveness endpoint was acute success (i.e., non-inducibility of all clinically relevant VTs).

**Results:**

Thirty-eight patients were enrolled with monomorphic VT (age 68 ± 12 years and 84% male), with an average of 1.7 ± 1.2 VTs targeted per patient. In total, 41 ± 23 RF applications per patient were delivered. Acute procedural success was 100% (95% CI, 91–100%). No primary safety endpoints were observed. Six-month follow-up was completed in 92% of patients with 81% (95% CI, 65–91%) freedom from sustained or treated VT. A repeat ablation was performed in three patients.

**Conclusions:**

Ablation of VT in SHD, using a temperature-controlled irrigated RF catheter, was safe and effective with a low rate of VT recurrence at 6 months.

**Graphical abstract:**

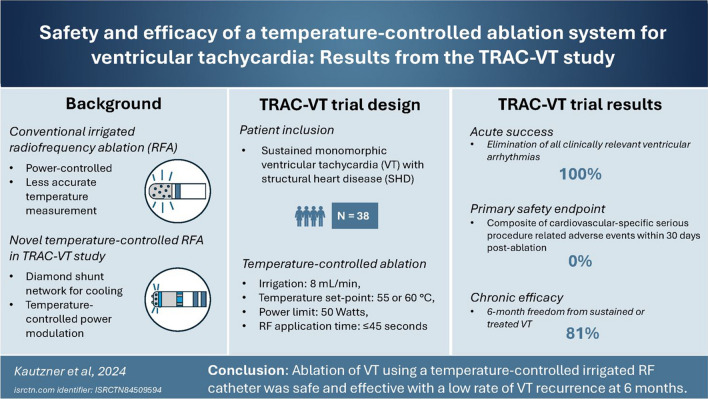

**Supplementary Information:**

The online version contains supplementary material available at 10.1007/s10840-025-01995-z.

## Introduction

Catheter ablation is a well-established treatment for patients with ventricular tachycardia (VT) [1, 2]. Currently, radiofrequency (RF) ablation catheters with open irrigation are predominantly used [3]. Importantly, open irrigation reduces the risk of complications such as char, steam pop, and thrombus formation that can arise with overheating at the catheter tip, but the saline irrigation limits the ability of conventional catheters to accurately measure tissue temperature [4]. Accurate thermal feedback at the catheter tip-tissue interface can provide important guidance for tailoring energy delivery which may in turn improve lesion efficacy while avoiding complications.

**Fig. 1:**
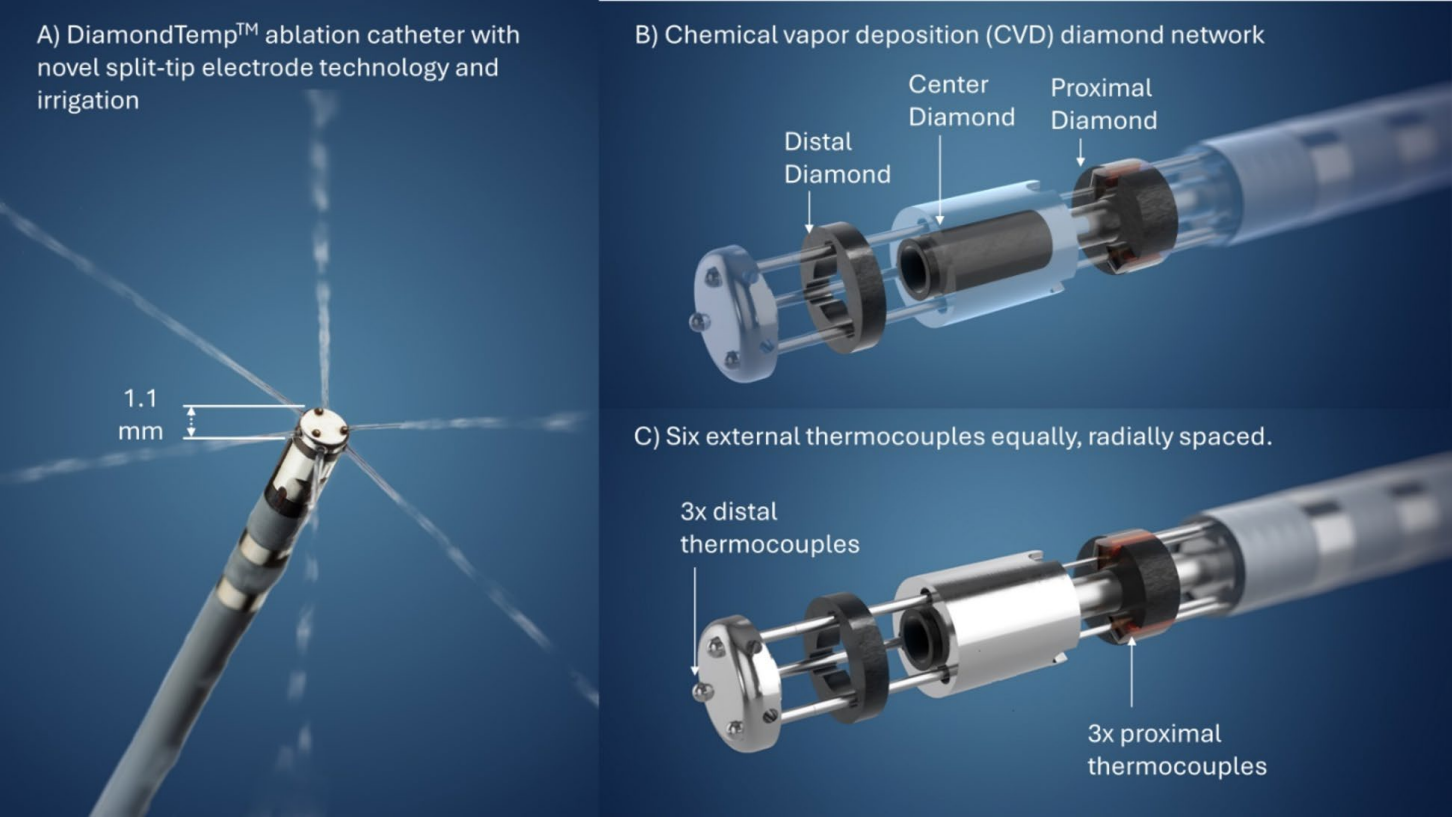
DiamondTemp catheter*.* The study evaluated the safety and efficacy of the DiamondTemp^TM^ ablation system (Medtronic, Inc.) for the treatment of patients with ventricular arrhythmias by catheter ablation

Accordingly, a novel RF ablation catheter (DiamondTemp ablation (DTA) catheter system, Medtronic, Minneapolis, MN; Fig. 1) has been designed to modulate power based on real-time temperature feedback from the tip-tissue interface with accuracy within 2–4 °C [5]. The system has been previously described in detail [6]. In brief, temperature is measured by six externalized thermocouples (three tip and three proximal RF electrodes) equally spaced around the catheter shaft. A network of (chemical vapor deposition) diamonds facilitates the rapid conduction of heat away from the catheter tip-tissue interface allowing the DTA system to use less saline irrigation from only six saline ports compared to traditional RF ablation catheters while improving the temperature fidelity at the thermocouples to accurately measure temperature throughout the application of RF energy. Furthermore, this system uses a distal split-tip circumferential electrode that provides real-time high-resolution electrograms and impedance recordings. These catheter characteristics are utilized to accurately monitor and maintain the tip-tissue temperature during RF energy delivery facilitating ablation in a temperature-controlled mode despite the use of open irrigation.

In the context of VT ablation, temperature-controlled irrigated RF ablation may be especially advantageous in reducing the risk of overheating during catheter ablation where longer application times may be required to achieve deeper lesions. We have previously reported pre-clinical data demonstrating the time-dependent association with lesion depth using the DTA system [7]. A prior feasibility study evaluated the DTA system for the treatment of VT in patients with ischemic cardiomyopathy (ICM) only (*n* = 10), reporting effective substrate modification with a high rate of acute procedural success (90%) and mid-term efficacy (88% reduction in total VT episodes at 6 months) [8]. Here, we further evaluate the safety and efficacy of the DTA system for VT ablation within a multicenter study in structural heart disease (SHD).

## Methods

### Trial design

TRAC-VT (isrctn.com identifier: ISRCTN84509594) was a prospective, multicenter, observational single-arm study enrolling patients at five hospitals in five European countries. The DTA system used in this study was market-approved in Europe at the time of enrollment. Ablation procedures were performed, and patients were followed for 12 months according to routine hospital practice and standard of care. Follow-up timepoints were protocol required at 3 and 6 months, and telephone follow-ups were required at 1 and 12 months post-ablation. Adverse events, changes in medication(s), and protocol deviations were assessed during all follow-ups. Additionally, at 3- and 6-month follow-up, a physical examination was performed, and cardiac arrhythmia status was assessed via 12-lead electrocardiogram, implantable cardioverter-defibrillator (ICD), or implantable cardiac resynchronization therapy defibrillator (CRT-D) device interrogation (including reading of VT events since prior follow-up).

### Study population

Subjects were enrolled with an indication for catheter ablation due to sustained monomorphic VT treated with a device shock or anti-tachycardia pacing (ATP) within the past 6 months in the presence of SHD. Patients with left ventricular ejection fraction (LVEF) less than 20%, recent stroke, myocardial infarction, or cardiac surgery were excluded from enrollment. A complete list of inclusion and exclusion criteria is found in Supplement Table 1. The Institutional Review Boards of each participating center reviewed and approved the study protocol. Written informed consent was obtained from all patients in accordance with the Declaration of Helsinki before enrollment.

**Table 1:** Baseline characteristics

	(*N* = 38)
Age (years)	67.6 ± 11.7
Male	32 (84.2%)
Body mass index	29.0 ± 5.8
Left ventricular ejection fraction (%)	35.9 ± 12.3
NYHA CLASS III/IV	10 (26.3%)
Months since initial VT diagnosis	6.5 ± 10.8
Prior ventricular tachycardia ablation	9 (23.7%)
Prior atrial fibrillation/atrial flutter ablation	2 (5.3%)
Symptomatic monomorphic ventricular ectopy	2 (5.3%)
Ventricular tachycardia non-sustained	9 (23.7%)
Ventricular tachycardia sustained monomorphic	38 (100%)
Ventricular fibrillation or flutter or polymorphic tachycardia	7 (18.4%)
Atrial fibrillation or flutter	15 (39.5%)
Underlying structural heart disease	
Myocardial infarction	31 (81.6%)
Dilated cardiomyopathy (DCM)	9 (23.7%)
Congestive heart failure	18 (47.4%)
Coronary artery disease	32 (84.2%)
Coronary artery bypass grafting	8 (21.1%)
Comorbidities	
Hypertension	21 (55.3%)
Diabetes	17 (44.7%)
Pulmonary disease	9 (23.7%)
Class I AAD failure	4 (10.5%)
Class II AAD failure	24 (63.2%)
Class III AAD failure	26 (68.4%)
Class IV AAD failure	1 (2.6%)
CRT-D/CRT-P/ICD implant	32 (84.2%)
Medication at enrollment	
Amiodarone	20 (52.63%)
Class III AAD	20 (52.63%)
Class I AAD	2 (5.26%)
Class I/III AAD	21 (55.26%)
Beta blocker (class II AAD)	36 (94.74%)
Digoxin (class V AAD)	2 (5.26%)
ACE inhibitors	10 (26.32%)
Statins	17 (44.74%)
Diuretic	28 (73.68%)
Anticoagulation	27 (71.05%)
Platelet aggregation inhibitor	13 (34.21%)
Direct oral anticoagulant	11 (28.95%)
Vitamin K antagonist	3 (7.89%)

### Treatment devices

The DTA generator is a temperature-controlled closed-loop system that modulates power to reach and sustain a programmable temperature set-point (default at 60 °C) and an irrigation pump that operates at 8 ml/min while RF energy is delivered, 4 ml/min nominal. The EnSite cardiac mapping system (Abbott, Inc., Chicago, IL) was used in all procedures to describe the myocardial substrate and navigate the DTA catheter.

In this study, RF ablation was performed in a temperature-control mode for all deliveries, and the temperature set-point was 55 °C or 60 °C, with power output limited to 50 W. RF energy was applied for a maximum of 45 s per target site. When considering a steam pop, the protocol required the catheter to be withdrawn with the tip electrode to be observed for the presence of char or coagulum formation.

### Study procedure

Conventional substrate-guided ablation was performed using the Ensite mapping system. In brief, voltage maps defined low voltage substrate during intrinsic rhythm and/or during right ventricular pacing and were compared with scar imaging data using intracardiac echocardiography (ICE) where available. Areas of late potentials and/or local abnormal ventricular activities (LAVAs) were identified with functional substrate mapping techniques used at investigator discretion to define slow conduction zones. Programmed electrical stimulation (PES) was performed to induce VT, and in cases of hemodynamically stable VT, entrainment mapping and VT activation mapping were employed to identify the critical components of the reentry circuit.

The ablation was performed to modify arrhythmogenic substrate, interrupt channels of slow conduction, and/or abolish late potentials or LAVAs. Following ablation, remapping of the substrate was performed to identify and eliminate residual substrate, and PES was performed to assess for the acute outcome. The PES protocol to evaluate acute success was based on local standards of care at each center using up to three extra-stimuli. Acute success was defined as non-inducibility of clinically relevant VT (excluding polymorphic VT, ventricular flutter, and ventricular fibrillation). Post-procedural antiarrhythmic medication maintenance was at the investigator’s discretion, and all patients received a minimum of 90-day oral anticoagulation.

### Trial endpoints

The primary effectiveness endpoint (acute success) was achieved when all clinically relevant VTs (episodes that were documented earlier and responsible for patient symptoms) were terminated and no longer inducible. Classification of clinical relevance of VT was at investigator discretion. The primary safety endpoint was defined as freedom from cardiovascular-specific serious, procedure-related adverse events occurring within 30 days post-ablation. Cardiovascular-specific was defined as the following composite of events, including cardiovascular-related death, cardiac tamponade or perforation, major bleed requiring a transfusion or resulting in a ≥ 20% fall in hematocrit (denoted bleeding complication), myocardial infarction, stroke, transient ischemic attack, thromboembolism, or pulmonary edema. All serious adverse events were adjudicated by an independent event committee consisting of cardiologists experienced in VT ablation.

Endpoints to further characterize the performance of the DTA system included chronic effectiveness (defined as the percentage of patients without documented sustained or treated VT which included arrhythmias that were faster than 100 beats/min for at least 30 s or treated by shock or ATP through 6 months post-ablation). The DTA system was further evaluated by assessing procedure-related endpoints, including the incidence of suspected steam pop formation, presence of char or coagulum formation, total number of RF ablations per procedure, total fluid infused through the assigned ablation catheter, and procedure times (including total procedure time, total RF treatment time, duration of RF ablations, and fluoroscopy time).

### Statistical methods

Analysis included all enrolled patients where the study device was inserted. Descriptive statistics were used to summarize baseline and procedural characteristics. Specifically, categorical variables were reported as counts and proportions and displayed as percentages (%). For continuous variables, mean ± standard deviations were reported. The primary objectives of assessing acute efficacy and acute safety were calculated using a proportion and 95% exact binomial confidence intervals. The long-term procedural efficacy results were evaluated using Kaplan–Meier curves and estimates of the survival with 95% confidence intervals using the log–log transformation. Analyses were conducted with R version 4.3.1 (R Core Team 2023) and SAS Software, Version 9.4 of the SAS System.

## Results

### Patients

Between June 2018 and December 2022, the study enrolled 38 subjects at five sites in five European countries. Baseline patient characteristics are shown in Table 1. Mean patient age was 68 ± 12 years with 84% being male. Nine patients had a prior VT ablation. There were two protocol deviations related to inclusion criteria: one patient with a history of sustained monomorphic VT did not have a documented episode during the 6 months before enrollment and one patient did not have confirmed SHD. All other inclusion and exclusion criteria were fulfilled**.**

### Procedure characteristics

General anesthesia was used in 32% of the patients. In the majority of cases, the substrate was defined by mapping in sinus rhythm or during right ventricular pacing. Entrainment mapping was performed in 32% of patients. Late potentials were targeted in 79%, LAVAs in 76%, and decrementing evoked potentials in 40% of patients. The average ablation time (i.e., time from first to last ablation) was 83.9 ± 42.8 min. RF energy was applied for an average of 25.1 ± 8.7 s with an average temperature of 50.6 ± 3.5 °C (with a power delivery of 43.4 ± 7.1 W) for each lesion. In total, a mean of 40 ± 23 RF applications per patient were performed. Total fluid infused through the assigned ablation catheter was 283.2 ± 124.8 ml. VTs originating from reentrant circuits were ablated in 83% of patients while the remaining patients had focal VT. Additional procedural information is found in Table 2.

**Table 2:** Procedure characteristics

	**VT** (*N* = 38)
Procedure time (min)	178.4 ± 65.0
Catheter dwell time (min)	84.0 ± 42.8
Time from first to last ablation (min)	83.9 ± 42.8
Application duration (s)	25.1 ± 8.7
Fluoroscopy time (min)	24.3 ± 17.0
Conscious sedation	26 (68.4%)
Hemodynamically tolerated VT	16 (69.6%)
Number of applications^a^	41.0 ± 22.6
Average power (W)	43.4 ± 7.1
Average temperature (°C)	50.6 ± 3.5
Maximum temperature (°C)	62.9 ± 4.2
Impedance drop (Ω)	− 13.1 ± 5.2
Fluid infused (ml)	283.2 ± 124.8
EnSite mapping used	38 (100%)
Patients with ventricular tachycardia targeted	35 (92.1)%
Number of VTs targeted/patient	1.7 ± 1.2
Number of VTs induced/patient	1.2 ± 0.5
Cycle length of treated VTs (ms)	330.1 ± 89
VT with focal origin	5 (16.7%)
Mapping	
Comparison of voltage map with scar imaging using ICE	18 (47.4%)
Annotation of late potentials on the electro-anatomical map	29 (76.3%)
Annotation of local abnormal ventricular activities on the electro-anatomical map	28 (73.7%)
Pacing to assess slow conduction	28 (73.7%)
Entrainment mapping	12 (31.6%)
Ablation targets	
Late potentials	30 (79.0%)
Local abnormal ventricular activities	29 (76.3%)
Decrementing evoked potentials	15 (39.5%)
Earliest EGM potentials/maximum precocity	3 (7.9%)
Acute procedural success (clinical VT not inducible)	38 (100%)
All VT not inducible	32 (84.2%)

^a^Excluding one patient with epicardial approaches

### Acute procedural success

The primary efficacy endpoint defined as acute procedural success was 100% (95% CI, 91–100%), and the average number of targeted VTs per patient was 1.7 ± 1.2. Targeted VTs had a mean cycle length of 330 ± 89 ms. After ablation therapy, all clinically relevant VTs were non-inducible, and in 84% of patients, no VT was inducible.

### Steam pops

Throughout the study, the steam pop rate was markedly low, 0.41% of all endocardial applications (6/1450), as shown in supplement Table 4. Steam pops occurred at four centers in four patients. The majority of patients with steam pops (three out of four) were either the first or second enrollment at the respective center. None of the steam pops resulted in an adverse event nor continuing patient sequela. In the RF generator files, no parameters could be identified that distinguished applications with versus without steam pops. Specifically, the surface temperature was at or above 60 °C in only one of six applications, and during four applications, the temperature was at or above 55 °C at any thermocouple. Also, application duration was between 11 and 45 s, and the impedance change ranged between 12 and 17 Ω. Upon suspicion of steam pop, per the protocol, the catheter was removed, and no char/coagulum was observed in any steam pop case. Unrelated to steam pop, char/coagulum was observed during one procedure. In this patient, a pseudoaneurysm at the vascular access site was adjudicated as procedure-related, but the event was not deemed a serious adverse event. No serious device-related adverse events were related to char or coagulum.

### Safety

There was no primary safety event (95% CI, 0.0–0.093%). Serious cardiovascular-specific events that were not procedure-related and not within 30 days after the initial ablation (and therefore not a primary safety event) occurred in two patients. The aforementioned patients had suffered from non-sudden cardiac deaths. Specifically, one patient expired at 51 days after index ablation after having a stroke 7 days earlier, and one patient expired at day 218 with pulmonary edema. A listing of all serious adverse events is found in the supplement Table 3. Serious adverse events adjudicated as possibly or causally related to the ablation procedure were reported in four patients (10.5%; 95% CI, 2.9–24.8%).

During the 12-month study follow-up, three patients died. The two previously mentioned deaths at days 51 and 218 were non-sudden cardiac deaths, and the third death occurred 287 days after the index ablation which was non-cardiac and secondary to respiratory failure. No deaths were adjudicated as device nor procedure-related events.

### Chronic efficacy

**Fig. 2:**
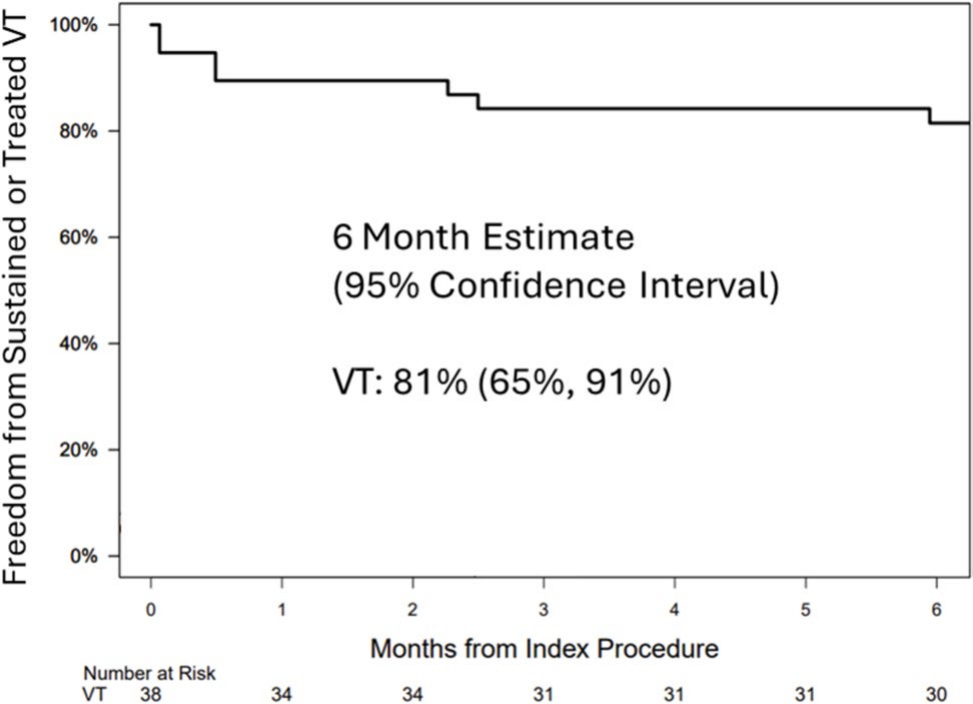
Chronic efficacy. Freedom from sustained (≥ 30 s) or treated VT

The 6-month follow-up was completed in 35 (92%) patients. Chronic success from sustained VT (≥ 30 s) or treated VT following 6 months was 81% (95% CI, 65–91%) as shown in Figure 2. Documented within 6 months were 65 sustained or treated VTs in seven patients with a mean cycle length of 348 ± 91 ms. In total, 58 of these episodes were treated by ATP and six with shock therapy. One VT terminated spontaneously after more than 30 s. Of the 7 patients with recurrent VT, three (43%) subjects underwent a repeat ablation procedure, with one patient requiring two repeat ablation procedures. All sustained episodes were documented in patients with an ICD or CRT-D device.

Regarding antiarrhythmic drug (AAD) usage, amiodarone was prescribed to 20 patients (53%) prior to the ablation procedure. No other class III AADs were prescribed. At hospital discharge, amiodarone was discontinued in two patients resulting in 18 patients (47%) discharged on amiodarone. Amiodarone was initiated in one patient within the 6-month follow-up period, and one patient on amiodarone died during the study follow-up period. At 6-month follow-up, 18 of 36 patients (50%) received amiodarone. Class I AADs were prescribed in two patients (5%) at enrollment (one who was also on amiodarone), in one patient (3%) at hospital discharge, and in two patients (6%) at 6-month follow-up (one of which was also on amiodarone). Beta blockers were given to 36 patients (95%) at enrollment, 37 patients (97%) at hospital discharge, and 34 patients (94%) at 6-month follow-up.

## Discussion

The main findings of the multicenter, prospective TRAC-VT study are the following data: (1) acute procedural success was achieved in all patients; (2) freedom from sustained VT or treated VT was 81% at 6 months; and (3) no primary safety endpoint events were observed.

After a first-in-human experience in a single-center study of ten patients [8], the TRAC-VT study is the first study assessing the safety and efficacy of a temperature-controlled irrigated RF ablation catheter for VT ablation in patients with both ICM and non-ischemic cardiomyopathy (NICM). The present study demonstrates excellent primary efficacy with 100% acute procedural success. This is an important observation since acute procedural success is associated with improved arrhythmia-free survival and all-cause mortality [9, 10]. Such rates of acute procedural success determined by VT non-inducibility endpoint testing are rarely achievable with conventional ablation technologies [10].

Chronic success from sustained or treated VT at 6 months was achieved in 81% of patients in the VT group as determined by device interrogations. Survival from recurrence of sustained VT in patients with SHD after 6 months varies from 77.5 to 91.5% (results estimated based on the published Kaplan–Meier curves) [11–14], and chronic success rates are dependent on underlying SHD with lower success rates observed in patients with NICM [1]. The sustained VT-free survival of 81% (95% CI, 65–91%) accomplished in this study falls into the range of these previously reported success rates, and the TRAC-VT result is therefore comparable with previously reported studies with a mix of ICM and NICM substrates.

For the safety analysis, no primary safety event was reported. This safety event rate is lower than the overall major complication rates previously reported in SHD patients undergoing catheter ablation for VT (3.8–11.2%) [2]. Furthermore, the rate of procedure- or device-related serious adverse events is lower than the rate reported in the recent LESS-VT trial [11]. Specifically, the LESS-VT trial evaluated the ablation of sustained monomorphic VT in patients with NICM using an irrigated RF catheter. This trial reported an overall procedure- or device-related serious adverse event rate of 21.1%, which is higher than the present study rate of 10.5% in the VT group. Importantly, within the TRAC-VT study, there were no deaths within 7 days of the index ablation, and there was no report of cardiovascular-related death, cardiac tamponade/perforation, bleeding complications, myocardial infarction, nor pulmonary edema related to the ablation procedure. Consequently, the TRAC-VT study demonstrates that the DTA irrigated RF ablation system can safely operate in a temperature-control mode during VT ablation without the use of contact force sensing data.

The first publication on VT ablation with DTA reported a steam pop (resulting in pericardial tamponade) with a lesion duration of 44 s, a maximum temperature of 60 °C, and an impedance reduction of 15 Ω [8]. Here, we reported steam pops during six applications from a total of 1450 endocardial applications (0.41%) without any procedure-related serious adverse events (nor surgical interventions). Our data on the rate of observed steam pops are in alignment with conventional power-controlled RF catheters (0.1 to 1.5%) [15, 16]. However, ablation parameters in applications with steam pops did not differ from the ablation parameters in the other (non-steam pop) applications within this TRAC-VT study. Importantly, tissue surface temperature can underestimate maximal tissue temperature, which is dependent on numerous factors including ablation duration, heat latency, tissue contact, current density, and tissue conductivity [16]. A learning curve may be involved when using the DTA catheter, as all four procedures (where steam pops were suspected) occurred at different centers predominately during the first or second procedural usage. Thus, further research is required to understand the mechanism of steam pops with traditional power-controlled and temperature-controlled RF catheters with the determination of measurable predictors to prevent them.

In summary, the TRAC-VT trial demonstrates that the DTA temperature-controlled RF delivery system is safe and effective for structural heart VT ablation. Further direct comparative studies to other ablation technologies may help elucidate the potential clinical benefits of a temperature-controlled RF ablation strategy for the treatment of ventricular arrhythmias.

## Limitations

This study included a non-randomized cohort with no comparative control population. As with the majority of clinical VT studies, the cohort is relatively small, and the data may not be representative of a larger global population of patients with VT. The PES protocol to evaluate acute success was based on local standards of care at each hospital and was not predefined within the study protocol. In three patients (7.9%), VT was not inducible by PES before the ablation and also after substrate modification. Evaluation of chronic success was limited by an absence of cardiac implantable electronic devices in 16% of patients (6/38). Antiarrhythmic medication prescription(s) was based on local standards and not defined within the study protocol. The use of amiodarone, having the PES protocol not predefined, and lack of ICD/CRT-D device interrogation data in a limited number of cases may have resulted in an overestimation of acute and chronic ablation success. Finally, the proportion of ICM versus NICM patients in this mixed study population may impact overall estimates of efficacy and safety, with NICM patients generally having a lower success rate and higher safety event rate compared to ICM patients.

## Conclusion

Ablation of VT using a temperature-controlled irrigated RF catheter was safe and effective with no primary safety event, 100% acute success, and low VT recurrence at 6-month follow-up.

## Supplementary Information

Below is the link to the electronic supplementary material.

Supplementary file1 (DOCX 30.6 KB)
