# Supplementary material for: Safety and efficacy of a temperature-controlled ablation system for ventricular tachycardia: Results from the TRAC-VT study
Source: J Interv Card Electrophysiol. 2025 Feb 1;68(6):1217–24. doi: 10.1007/s10840-025-01995-z (PMC12399702; doi:10.1007/s10840-025-01995-z)
Supplement: Supplementary file 1 — Supplementary file1 (DOCX 30.6 KB) [file 10840_2025_1995_MOESM1_ESM.docx]

**Supplement**

**Table 1** Participating Sites and Investigators

| **Investigator** | **Site, City, Country** | **Enrolments:** |
| --- | --- | --- |
| Prof. Josef Kautzner | Institut klinické a experimentální medicíny (IKEM), Prague, Czech Republic | 14 |
| Dr. Javier Moreno Planas | University Hospital Ramon y Cajal, Madrid, Spain | 5 |
| Prof. Claudio Tondo | Università degli Studi di Milano, Centro Cardiologico Monzino, Milano, Italy | 1 |
| Prof. Tarvinder Dhanjal | University Hospitals Coventry & Warwickshire, Coventry, UK | 16 |
| Prof. Frédéric Anselme | CHU de Rouen, Cardiologie, Rouen, France | 2 |
|  | **Total** | **38** |

**Table 2** Inclusion and Exclusion Criteria

| **Inclusion Criteria:** (Candidates must meet ALL of the following criteria to be enrolled in the TRAC-VT Study)   1. Above eighteen (18) years of age or minimum age as required by local law 2. Suitable candidate for catheter ablation 3. Patients with at least one (1) documented episode of sustained VT meeting all criteria (a-d) below:    1. Episode occurring within previous 6 months    2. Episode must be monomorphic    3. Episode requiring external cardioversion or ICD anti-tachycardia pacing (ATP) or shocks    4. Structural heart disease with ischemic or non-ischemic dilated cardiomyopathy 4. Patient failed any anti-arrhythmic drug (AAD) regime unless contraindicated or not tolerated 5. Patient is willing and able to provide written consent. |
| --- |
| **Exclusion Criteria:**  (Candidates will be excluded from the TRAC-VT Study if any of the following conditions apply at the time of enrollment or procedure)   1. Contraindication to catheter ablation 2. Ventricular tachycardia due to transient, reversible causes 3. Exclusively polymorphic VT 4. Electrolyte imbalance 5. Use of left ventricular assist device (LVAD) or circulatory assist devices 6. Stroke (<6 months) 7. Presence of a left atrial or ventricular thrombus 8. Severe cerebrovascular disease 9. Active gastrointestinal bleeding 10. Unstable angina 11. Renal failure (on dialysis or at risk of requiring dialysis) 12. Active infection or fever 13. Currently NYHA Functional Class IV heart failure 14. Left Ventricular Ejection Fraction (LVEF) <20% 15. Myocardial infarct or previous cardiac surgery (<3 months) 16. Prosthetic mitral or aortic valve 17. Mitral or aortic valvular disease requiring immediate surgical intervention 18. Active ischemia who are eligible for revascularization 19. Contraindication to heparin 20. Thrombocytopenia or coagulopathy 21. Uncontrolled diabetes needing therapy 22. Pregnancy or women of child-bearing potential 23. Unable to give informed consent 24. Unable to attend follow-up visits 25. Life expectancy <12 months based on medical history or the medical judgement of the investigator. 26. Enrollment in a concurrent clinical study that in the judgement of the investigator would impact study outcomes 27. Acute or chronic medical condition that in the judgment of the investigator would increase risk to the patient or deem the patient inappropriate to participate in the study |

*^1^ The original inclusion criteria included ischemic or non-ischemic dilated cardiomyopathy. However, upon data review 86% of the patients did not fulfill this criterion. Therefore, the manuscript inclusion criteria include the criteria that were fulfilled with exclusion of dilated cardiomyopathy.*

**Table 3** Serious Adverse Events per Protocol Definition

| **Event ID** | **Duration from Index Ablation [days]** | **Serious Adverse Event** | **Classification^1^** |
| --- | --- | --- | --- |
| 1.1 | 29 | VT Recurrence |  |
| 1.2 | 81 | VT Recurrence |  |
| 1.3 | 301 | Angina Pectoris Progression |  |
| 2.1 | 2 | VT Recurrence |  |
| 2.2 | 1 | Dysuria | Possible Procedure Relation |
| 2.3 | 51 | Death due to cardiac failure | Non-sudden cardiac death |
| 2.4 | 44 | Stroke | Stroke |
| 3.1 | 76 | VT Recurrence |  |
| 4.1 | 17 | Heart Failure |  |
| 4.2 | 39 | Severe Anemia |  |
| 4.3 | 39 | Colon Sessile Polyp |  |
| 4.4 | 46 | Fever |  |
| 5.1 | 6 | Pocket Hematoma After Device Up-Grade | Bleeding Complication |
| 5.2 | 122 | Left Femur Subcapital Fracture |  |
| 5.3 | 122 | Impaired Kidney Function |  |
| 6.1 | 45 | VT Recurrence |  |
| 6.2 | 69 | Incessant VT / electrical Storm | Possible Procedure Relation |
| 6.3 | 88 | Incessant VT / electrical Storm |  |
| 6.4 | 110 | Sepsis of Unknown Origin |  |
| 6.5 | 110 | Incessant VT / ectrical Storm |  |
| 6.6 | 110 | Multi-Organ-Failure |  |
| 7.1 | 0 | Induction of VTs of various morphologies during ablation procedure | Causal Procedure Relation |
| 8.1 | 32 | Hospital admission for dizziness |  |
| 8.2 | 337 | Septic shock |  |
| 8.3 | 337 | Streptococcus Dysgalactiae & E. coli bacteria |  |
| 8.4 | 354 | Admission due to back pain due to L3 vertebral fracture |  |
| 8.1 | 0 | Femoral bleeding from vascular access^2^ | Causal Procedure Relation |
| 9.1 | 76 | VT post ablation |  |
| 9.2 | 218 | Death due to pulmonary oedema | Non-sudden cardiac death; Pulmonary Edema |
| 10.1 | 200 | Palpitations |  |
| 10.2 | 200 | Chest Pain |  |
| 11.1 | 246 | Monomorphic VT |  |
| 11.2 | 275 | Acute Kidney Infection |  |
| 11.3 | 287 | Death due to respiratory failure | Non-cardiac death |

*^1^Classification by an independent event committee: serious adverse event; device- / procedure relatedness; cardiovascular-specific event (cardiovascular-related death, sudden death, cardiac tamponade or perforation, bleeding complication, myocardial infarction, stroke, transient ischemic attack, thromboembolism, pulmonary edema).*

*^2^Consistent with major complication*

**Table 4** Applications with Steam Pop(s) during Index Ablation

| **Event ID** | **Max Temperature [°C]** | **Time after reaching 60°C [sec]** | **Duration [sec]** | **Minimum RF Impedance [Ω]** | **Impedance Drop [Ω]** | **Location** | **Visual confirmation by intracardiac echocardiography** |
| --- | --- | --- | --- | --- | --- | --- | --- |
| 1.1 | 53.3 | 0 | 13 | 84 | 12 | LV inferior wall | Yes |
| 1.2 | 52.7 | 0 | 11 | 82 | 12 | LV inferior wall | Yes |
| 1.3 | 55.9 | 0 | 19 | 81 | 13 | LV inferior wall | Yes |
| 2.1 | 59.9 | 0 | 22 | 71 | 17 | Inferior lateral right Ventricular outflow tract | No |
| 3.1 | 48.2 | 0 | 45 | 88 | 13 | Left outflow tract region | No |
| 4.1**^1^** | 61.3 | 1 | 15 | 105 | 39 | Epicardial, antero-apical aspect of the LV | No |
| 5.1 | 61.1 | 4 | 39 | 80 | 15 | LV septum | No |

*^1^Patient was excluded from analysis on steam pops due to the epicardial ablation approach*
